# Leptin as a predictor of metabolic syndrome in prepubertal children

**DOI:** 10.1590/2359-3997000000199

**Published:** 2016-08-31

**Authors:** Isabel Madeira, Maria Alice Bordallo, Nádia Cristina Rodrigues, Cecilia Carvalho, Fernanda Gazolla, Paulo Collett-Solberg, Clarice Medeiros, Ana Paula Bordallo, Marcos Borges, Claudia Monteiro, Rebeca Ribeiro

**Affiliations:** 1 Faculdade de Ciências Médicas Departamento de Pediatria Universidade do Estado do Rio de Janeiro Rio de Janeiro RJ Brasil Faculdade de Ciências Médicas, Departamento de Pediatria, Universidade do Estado do Rio de Janeiro (UERJ), Rio de Janeiro, RJ, Brasil; 2 Faculdade de Ciências Médicas Departamento de Medicina Interna UERJ Rio de Janeiro RJ Brasil Faculdade de Ciências Médicas, Departamento de Medicina Interna, UERJ, Rio de Janeiro, RJ, Brasil; 3 Faculdade de Ciências Médicas Departamento de Tecnologias da Informação e Educação em Saúde UERJ Rio de Janeiro RJ Brasil Faculdade de Ciências Médicas, Departamento de Tecnologias da Informação e Educação em Saúde, UERJ, Rio de Janeiro, RJ, Brasil; 4 Instituto de Nutrição Departamento de Nutrição Aplicada UERJ Rio de Janeiro RJ Brasil Instituto de Nutrição, Departamento de Nutrição Aplicada, UERJ, Rio de Janeiro, RJ, Brasil; 5 Hospital Universitário Pedro Ernesto Unidade Docente Assistencial de Endocrinologia e Metabologia UERJ Rio de Janeiro RJ Brasil Hospital Universitário Pedro Ernesto, Unidade Docente Assistencial de Endocrinologia e Metabologia, UERJ, Rio de Janeiro, RJ, Brasil; 6 Faculdade de Ciências Médicas UERJ Rio de Janeiro RJ Brasil Faculdade de Ciências Médicas, UERJ, Rio de Janeiro, RJ, Brasil

**Keywords:** Children, ROC curve, leptin, obesity, insulin resistance

## Abstract

**Objective:**

Leptin has been suggested as a potential biomarker of cardiovascular risk. This paper aims to ascertain, based on a sample of prepubertal children, which serum leptin value best suited to identify metabolic syndrome (MS).

**Subjects and methods:**

This observational, cross-sectional study recruited children from the outpatient pediatrics clinic, with the purpose of validating serum leptin level cutoffs to identify MS. All obese and overweight children who met eligibility criteria were included in the study, as was a sample of normal-weight children. The sample underwent clinical assessment and blood fasting glucose, lipid profile, insulin, and leptin were measured. Sensitivity and specificity were estimated for each leptin measurement, using MS as the outcome. These values were used to construct a receiver operating characteristic (ROC) curve. The association between MS and leptin was assessed using logistic models to predict MS.

**Results:**

A total of 65 normal weight, 46 overweight, and 164 obese children were analyzed (160 boys, 115 girls; age: 93.7 ± 17.8 months). The most appropriate leptin cutoff was 13.4 ng/mL (sensitivity 67.6%; specificity 68.9%; accuracy 72.1%). The logistic model indicated that leptin levels above 13.4 ng/dL were significantly associated with MS and that, for every 1 ng/dL increase in leptin levels, the odds of MS increase by 3% (p = 0.002; OR 1.03; 95% CI 1.01-1.05).

**Conclusions:**

Leptin may be a useful biomarker of cardiovascular risk in prepubertal children, with an optimal cutoff of 13.4 ng/mL. Identification of potential new risk markers for cardiovascular disease in children could contribute to the development of preventive strategies.

## INTRODUCTION

Obesity is currently a highly prevalent condition, including in Brazil ( 1 , 2 ). The most significant complication of obesity, atherosclerotic cardiovascular disease, now constitutes the leading cause of death in adults in the western world. The main risk factors for cardiovascular disease are obesity, hypertension, dyslipidemia, and type 2 diabetes mellitus (T2DM), which together compose the so-called metabolic syndrome (MS). In this syndrome, insulin resistance and hyperinsulinemia would explain the core role of obesity and its association with the other abnormal phenomena observed. The effects of these factors appear to begin in childhood ( 3 ).

The pathophysiology of obesity involves an imbalance between energy intake and energy expenditure. Several neuroendocrine factors have been implicated in this energy imbalance, such as adipocytokines, proteins produced by adipose tissue. One of the most important adipocytokines is leptin.

This hormone signals, through central pathways, a decrease in food intake and increase in energy expenditure, in addition to having peripheral actions. In muscle, leptin stimulates fatty acid oxidation by activating adenosine monophosphate kinase. It also removes lipids from non-adipose tissue, preventing lipotoxicity, possibly due to its ability to block stearoyl-coenzyme A desaturase, and inhibits hepatic triglyceride buildup by activating phosphatidylinositol 3-kinase ( 4 ).

Circulating leptin levels correlate with body adiposity in adults and children ( 5 ), and the high leptin levels found in obese individuals are believed to indicate leptin resistance ( 6 ). Furthermore, studies in children have shown that high leptin levels correlate with greater fat mass growth over time ( 7 ). Although most obese children have high leptin levels, mutations in the leptin receptors are rare. This adipokine is believed to cross the blood–brain barrier by means of a saturable transport system, which would limit its uptake by central receptors ( 6 ).

Other aspects related to leptin have been assessed in the pathophysiology of obesity. Leptin may contribute to insulin resistance and its metabolic correlates and appears to have a direct pro-thrombotic effect, in addition to acting synergistically with insulin and free fatty acids to stimulate sympathetic activity and vasoconstriction ( 8 ). Hence, leptin and insulin interact to modulate vascular function, and this interaction may have major implications in the vascular dysfunction of MS.

In the pediatric age range, obesity appears to be an important trigger of insulin resistance ( 3 ), which makes obese children a high-risk group and has led investigators to search for clinical and laboratory indicators in this population. Nevertheless, there is no consensus definition of MS in children. A review on the topic found 40 different definitions adapted from those proposed for adults ( 9 ). In 2007, the International Diabetes Federation (IDF) proposed the latest definition of MS for children over the age of 10 years, based on the presence of increased waist circumference plus two of the following elements: hypertriglyceridemia; low HDL cholesterol; hypertension; and impaired fasting glucose or T2DM ( 10 ). A systematic review on MS in children found 26 studies adopting this definition ( 11 ).

In a recently published study, our group demonstrated that leptin is positively associated with insulin resistance in prepubertal children after adjusting for sex, age, and body mass index (BMI) Z-score ( 12 ). Other authors reported similar results in children ( 13 , 14 ), suggesting a role of leptin as a potential modulator of glucose metabolism and insulin resistance, regardless of obesity. These studies highlight the importance of leptin as a cardiovascular risk marker in this age group.

Identification of potential new risk markers for cardiovascular disease in children could contribute to the development of early intervention strategies, particularly preventive ones. However, no published studies have proposed serum leptin level cutoffs for the pediatric population.

Within this context, the objectives of this study are to ascertain, within a case series of prepubertal children with normal and excess weight, which serum leptin value best suited to identify MS, and to evaluate the association between leptin and MS.

## SUBJECTS AND METHODS

This observational, cross-sectional study recruited children from the outpatient general pediatrics clinic of Hospital Universitário Pedro Ernesto da Universidade do Estado do Rio de Janeiro (HUPE-UERJ), a teaching hospital in Rio de Janeiro, Brazil, with the purpose of validating serum leptin level cutoffs to identify MS. All children aged 5–11 years who were prepubertal, overweight or obese, otherwise healthy, and were not taking part in any weight loss program were invited to take part in the study. All eligible children who met these criteria were included. Normal-weight, healthy, prepubertal children, matched by age, from the same Pediatric well-child care clinic were recruited as controls, selected in a first-come first-serve order from May 2008 to December 2011. Study sample size, 275 children, was considered sufficient to achieve statistical power of 80%, with a level of significance set at 5%, for an error of 5%, based on the total population of children seen at the clinic ( 15 ).

The children recruited for the study underwent a complete clinical assessment. Weight was measured with the participants barefoot and wearing minimal clothing, on a Filizola scale (Filizola, São Paulo, SP, Brazil) with a resolution of 100 g. Height was measured with a Harpenden-type wall-mounted stadiometer (Tonelli, Criciúma, SC, Brazil) with a resolution of 1 mm. Waist circumference was measured at just above the uppermost lateral border of the right ilium, at the end of a normal expiration, as recommended in the Third National Health and Nutrition Examination Survey (NHANES III) Anthropometry Procedures Manual ( 16 ), using a Mabbis^®^ Gulick-type tape measure (Cardiomed, Curitiba, PR, Brazil).

Blood pressure was measured in the right arm using the auscultatory method, with each participant in the sitting position and at rest, using a Tycos^®^ aneroid sphygmomanometer (Welch Allyn Company, Arden, DE, USA) with cuffs of appropriate size.

Blood was collected for laboratory testing after a 12-hour fast. Glucose, total cholesterol, HDL cholesterol, and triglycerides were measured in a Konelab analyzer with the BT 3000 Winer kit, which employs the following assay methods: for glucose, the GOD-PAP (oxidase) enzymatic method; for cholesterol, the CHOP-POD (esterase/oxidase) enzymatic method; for triglycerides, the GPO-PAP (oxidase) enzymatic method; and for HDL cholesterol, the enzymatic colorimetric method (Winterlab, Rosario, Santa Fe, Argentina).

Insulin was measured in a Gamma-C12 counter using the Coat-A-Count solid-phase ^125^I-labeled radioimmunoassay (DPC, Los Angeles, CA, USA). The intra-assay and inter-assay coefficients of variation were 3.1–9.3% and 4.9–10.0% respectively.

Leptin was also measured in the Gamma-C12 counter, using the double antibody PEG radioimmunoassay method, with a kit that uses ^125^I-labeled human leptin and human leptin antiserum (Linco Research, St. Charles, MO, USA). The intra-assay and inter-assay coefficients of variation were 3.4–8.3% and 3.0–6.2% respectively.

The HOMA-IR score was calculated by multiplying the fasting blood glucose (in mmol/L) by the fasting insulin level (in µIU/mL) and dividing the product by 22.5, as noted elsewhere ( 17 ).

Obesity, overweight, and normal weight were defined using the BMI for sex and age standards proposed by the World Health Organization (WHO). The criteria are as follows: a body mass index (BMI) Z-score greater than +2 denotes obesity; greater than +1 and less than or equal to +2, overweight; and greater than or equal to -2 and less than or equal to +1, normal weight ( 18 ).

The definition of MS was adapted from the IDF proposal for children over the age of 10 years ( 10 ), and waist circumference was defined as increased when the measurement was within or above the 90^th^ percentile for sex and age in the NHANES III table that combines children of African-American, European-American, and Mexican-American ethnicity ( 16 ). Hypertension was defined according to the criteria recommended in the 1^st^ Brazilian Guideline on the Prevention of Atherosclerosis in Childhood and Adolescence ( 19 ). The cutoff points adopted for fasting blood glucose, HDL cholesterol, and triglycerides were those recommended by the same guideline: impaired fasting glucose, values ≥ 5.6 mmol/L (100 mg/dL); low HDL cholesterol, values < 1.16 nmol/L (45 mg/dL); and increased triglycerides, values ≥ 1.46 mmol/L (130 mg/dL) ( 19 ).

The collected data were entered into Excel 7 spreadsheets (MapInfo Corporation, Troy, NY, USA) and analyzed in R-Project 3.0.1 (Free Software Foundation, Boston, MA, USA).

Simple and multivariate logistic models, the latter adjusted for sex and age and both having serum leptin levels as the main predictor, were used to predict MS. The results obtained were used to construct two receiver operator characteristic (ROC) curves.

The present study was approved by the HUPE-UERJ Research Ethics Committee with protocol no. 173-CEP/HUPE–CAAE; 0020.0.228.000-07. It is also registered with the Brazilian National Research Ethics Commission under number 127374.

## RESULTS

Table 1 describes the clinical and metabolic profile of study participants stratified by nutritional status is provided. A comparison between the MS and no MS groups is shown in Table 2 .

**Table 1:** Profile of study participants stratified by nutritional status

Parameter	Obese children	Overweight children	Normal weight children	P-value
Participants, n (%)	164 (59.6)	46 (16.7)	65 (23.6)	
Age in months	94.68 ± 17.70	92.93 ± 18.68	91.85 ± 17.51	0.52
Sex				
Male	104 (63.4)	21 (45.7)	35 (53.8)	0.07
Female	60 (36.6)	25 (54.3)	30 (46.2)	
Metabolic syndrome	33 (97.1)	1 (2.9)	0 (0)	0.00001
Total cholesterol in mg/dL^‡^	166.43 ± 30.78	166.22 ± 30.36	153.52 ± 33.75	0.02
HDL cholesterol in mg/dL^§^	41.90 ± 9.00	52.48 ± 12.89	49.15 ± 11.70	0.00001
LDL cholesterol in mg/dL^‡^	103.90 ± 28.67	98.22 ± 30.64	89.82 ± 30.17	0.001
Triglycerides in mg/dL^§^	102.96 ± 55.35	77.96 ± 30.08	72.86 ± 28.40	0.00001
HOMA-IR^†^	2.15 ± 1.83	1.67 ± 1.02	0.78 ± 0.67	0.00001
Glucose in mg/dL	86.64 ± 8.72	86.78 ± 7.52	84.45 ± 7.21	0.16
Leptin in ng/mL*	18.6 ± 14.47	9.64 ± 9.05	3.29 ± 2.83	0.00001

Data expressed as absolute and relative frequencies (%) or mean ± standard deviation. Tukey test: * showed significant difference between all categories (obese, overweight, and normal weight children); ^†^ showed significant difference between obese and normal weight children and between overweight and normal weight children; ^‡^ showed significant difference between obese and normal weight children; ^§^ showed significant difference between obese and normal weight children and between obese and overweight children.

**Table 2:** Profile of study participants stratified by metabolic syndrome status

Parameter	Metabolic syndrome	No metabolic syndrome	P-value
Participants, n (%)	34 (12.4)	241 (87.6)	
Age in months	103.17 ± 18.6	92.4 ± 17.3	0.0009
Sex			
Male	23 (67.6)	137 (56.8)	0.23
Female	11 (32.4)	104 (43.2)	
BMI z-score^a^	3.54 ± 1.19	1.96 ± 1.77	0.00001
Total cholesterol in mg/dL	175.6 ± 25.1	161.6 ± 32.3	0.02
HDL cholesterol in mg/dL	36.8 ± 4.6	46.6 ± 11.4	0.00001
LDL cholesterol in mg/dL	103.6 ± 24.7	99.1 ± 30.5	0.41
Triglycerides in mg/dL	176.5 ± 62.4	79.7 ± 31.4	0.00001
HOMA-IR	3.3 ± 2.7	1.5 ± 1.2	0.00001
Glucose in mg/dL	88.76 ± 7.89	85.78 ± 8.21	0.046
Leptin in ng/mL	21.2 ± 11.3	12.4 ± 13.5	0.0004

Data expressed as absolute and relative frequencies (%) or mean ± standard deviation.

^a^ Z-score for body mass index.

Waist circumference was above the 90^th^ percentile for age in 135 (49.1%) children. Hypertension was detected in 4 (1.45%) children, for a prevalence of 8.82% (n = 3) in the MS group *versus* 0.41% (n = 1) in the no MS group. Impaired fasting glucose was observed in 4 (1.45%) children, all of whom were obese, for a prevalence of 2.94% (n = 3) in the MS group *versus* 1.24% (n = 1) in the no MS group.

**Figure 1:**
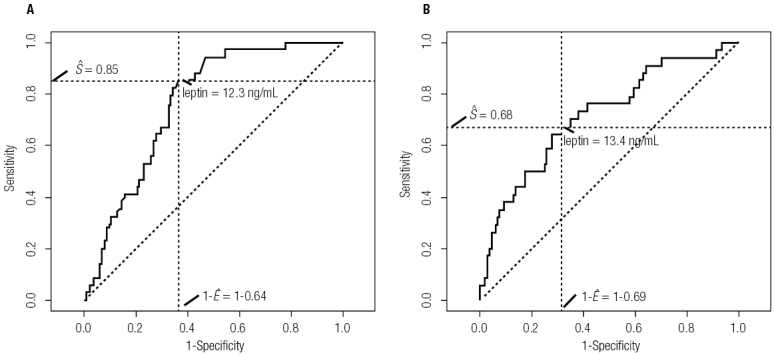
ROC curves of logistic regression prediction models for metabolic syndrome (response variable) in relation to the main predictor (leptin level), based on observations of prepubertal children. (A) Crude model: best cutoff point, 12.3 ng/mL; area under the curve, 76.4%; Youden’s index, 48.8%. (B) Model adjusted for sex and age: best cutoff point, 13.4 ng/mL; area under the curve, 72.1%; Youden’s index, 36.5%. On both charts, the best cutoff is denoted by the intersection of the dotted lines. The area under the curve represents the overall accuracy of the test.

Figure 1 shows two ROC curves plotted from the sensitivity and specificity values found for each leptin level measured in the study sample, with MS as the outcome. Curve A represents the simple logistic model for prediction of MS. In this model, a leptin value of 12.3 ng/mL (85% sensibility; 64% specificity) corresponded to the shoulder of the curve and had the best Youden’s index. Curve B represents the multiple logistic model, adjusted for sex and age. In this model, a leptin value of 13.4 ng/mL (68% sensibility; 69% specificity) corresponded to the shoulder of the curve and had the best Youden’s index.

**Figure 2:**
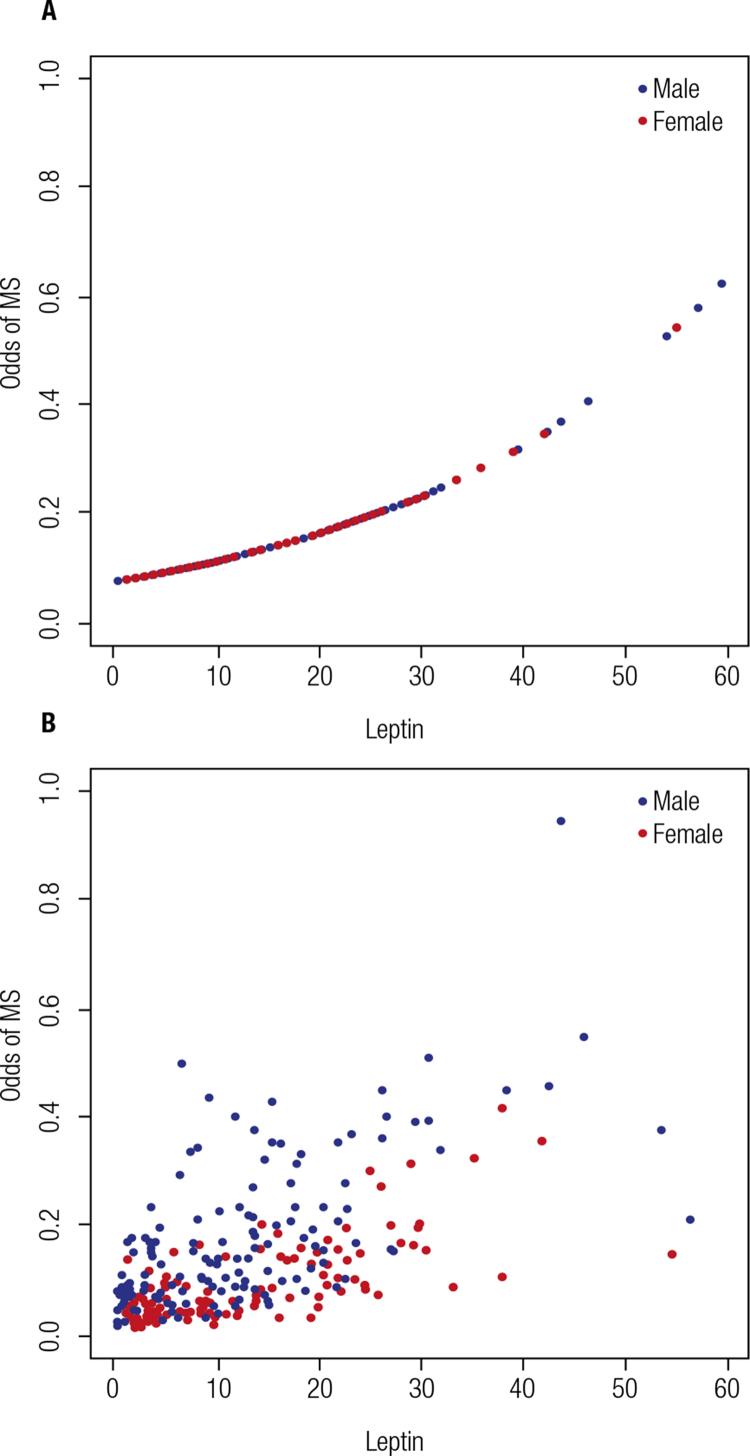
Odds of metabolic syndrome stratified by leptin levels (ng/mL). (A) Crude logistic model: odds ratio (95%CI) = 1.04 (1.01-1.06); p < 0.002; equation for the odds of metabolic syndrome = exp (- 2.51 + 0.03 * leptin). (B) Model adjusted for sex and age: odds ratio (95%CI) = 1.03 (1.01-1.06); p < 0.002; equation for the odds of metabolic syndrome = exp (-5.19 + 0.03 * leptin – 0,32 * sex + 0.03 * age in months).

In the multiple logistic model for prediction of MS, adjusted for sex and age, a leptin level above 13.4 ng/dL was significantly associated with MS (p = 0.002). Figure 2 shows the odds for MS for each leptin value, according to simple and multiple logistic models.

## DISCUSSION

The study sample was a very young group of children, and the substantial prevalence of metabolic syndrome according to the adapted IDF criteria is a concerning finding. We decided to use these criteria because they are currently the only standard for diagnosis of the syndrome in children and are recommended by authors who advocate that the use of unified criteria would contribute to the development of studies on the topic ( 11 , 20 ). However, in view of the lack of scientific evidence for this classification in under-10 children, these criteria should only be used in clinical practice in patients who are aged 10 years or older ( 10 ).

Despite the lack of a consensus definition for MS in children and the fact that the syndrome is not a disease in itself, but rather a constellation of risk factors for cardiovascular disease, its presence in children has been reported and investigated. According to a recent systematic review, the prevalence of MS is nearly 3.3% in the whole pediatric population, 11.9% in the overweight pediatric population, and 29.2% in the obese pediatric population ( 11 ). Longitudinal studies have shown that children with MS components grow into adults with MS, and that adolescents with the syndrome are at greater risk of premature cardiovascular disease in adulthood ( 21 ). This evidence justifies the search for new biomarkers of cardiovascular risk in children with excess weight, so as to identify those at the greatest risk, understanding the concept of biomarker as an indicator of pathogenic process ( 22 ).

Leptin is one such potential biomarker ( 23 ). In children, as in adults, its circulating levels correlate strongly with body fat ( 5 , 22 ), and high leptin levels are associated with greater fat mass growth over time ( 7 ) and with difficulty losing weight ( 24 ).

In a previous study conducted in the same population of the present study, leptin levels were positively associated with insulin resistance after adjusting for sex, age, and BMI Z-score ( 12 ).

Other authors reported similar results in studies of prepubertal children with normal and excess weight ( 13 , 14 ), suggesting a role of leptin as a potential modulator of glucose metabolism and insulin resistance, regardless of obesity.

Knowledge of the role of leptin within this panorama has grown to a point where some authors regard it as a programming factor for future development of obesity and its correlates, possibly via epigenetic mechanisms ( 25 ).

Within this perspective, the present study ascertained that, in a sample of prepubertal children with normal and excess weight, a circulating leptin level of 13.4 ng/mL was the optimal cutoff point to identify MS. The sensitivity and specificity of this cutoff were 68% and 69% respectively, which means that its use in prepubertal children will lead to a correct diagnosis of MS in approximately two-thirds of cases and correctly rule out the syndrome in just over two-thirds of children who do not have it.

Use of this cutoff revealed an association between leptin and MS after adjusting for sex and age. The adjusted logistic model showed that, for every 1-ng/dL increase in leptin levels, the odds of MS increase by 3% (p < 0.002).

This association has been described before by other authors, including González and cols., who recruited a randomized sample of 12-to-17-year-olds ( 26 ). Papoutsakis and cols., using the IDF definition of MS in a cohort of 1,138 healthy subjects (normal-weight, overweight, and obese) with a mean age of 11.2 years, demonstrated that leptin is a predictor of the number of metabolic syndrome components present ( 27 ). Pedrosa and cols., using the definition of MS proposed by the National Cholesterol Education Program Adult Treatment Panel III (NCEP ATP-III), showed that presence of the syndrome was associated with high leptin levels in overweight and obese children aged 7 to 9 years ( 28 ).

The novelty of the present study lies in the age range of the recruited participants. Other studies on this topic in young children are scarce, as most authors include adolescents in their series.

In view of their inherent peculiarities, prepubertal children must be studied separately from pubertal and postpubertal subjects, in whom the effects of sex steroids are already present. It has been established that insulin levels and the frequency of insulin resistance increase as puberty progresses ( 29 ). The effects of sex steroids are also reflected in leptin levels, which increase during puberty as well ( 26 ). Although studies enrolling prepubertal children exclusively are rare, it is known that some risk factors for cardiovascular disease are already present at this age, as shown in our investigation. This justifies further research into potential new biomarkers of cardiovascular risk in childhood, despite the technical difficulties inherent to study of such young subjects.

Now, more than ever, a consensus definition of MS is required ( 9 ). Development of such a definition requires proper definition of its components and of cutoffs for better identification of children at increased cardiovascular risk.

The limitations of the present study were mainly those imposed by the young age of the participants, as there is no consensus definition of the MS in this age range. In addition, some cardiovascular risk markers representative of MS, such as hypertension, impaired fasting glucose, and T2DM, are rare in children ( 23 , 30 ), a finding confirmed in the present case series.

Another limitation was the areas under the curves of the ROC curves ( Figure 1 ). Good values are 80-90, while cutoffs 70-80 are considered reasonable ( 15 ).

Study of the behavior of potential new biomarkers of cardiovascular risk in childhood may facilitate strategies for prevention and early intervention. Children with high leptin levels constitute a population to which resources and research efforts could be directed. However, due to the dearth of studies in this age group, we must stress that leptin measurement is still not applicable to pediatric clinical practice. Therefore, caution is warranted when attempting to identify young children at a supposedly increased risk of cardiovascular disease. In this age group, the most suitable approach would be to continue focusing on prophylaxis, i.e., promoting a healthy lifestyle.
